# Behavioral Changes in Cats Following Deslorelin Implantation or Surgical Gonadectomy †

**DOI:** 10.3390/vetsci12050430

**Published:** 2025-04-30

**Authors:** Mihaela Velkovska, Maria Pereira, Maja Zakošek Pipan, Darja Pavlin, Lluis Ferré-Dolcet, Simona Normando, Stefano Romagnoli

**Affiliations:** 1 Clinic for Reproduction and Large Animals, Veterinary Faculty, University of Ljubljana, 1000 Ljubljana, Slovenia; mv0087@student.uni-lj.si (M.V.);; 2 Department of Animal Medicine, Production and Health, University of Padova, 35020 Legnaro, Padova, Italy; 3 Clinic for Small Animals, Veterinary Faculty, University of Ljubljana, 1000 Ljubljana, Slovenia; 4 Anicura Ars Veterinaria, 08034 Barcelona, Spain; 5 Department of Comparative Biomedicine and Food Science, University of Padova, 35020 Legnaro, Padova, Italy

**Keywords:** felines, flare-up, pharmacological sterilization, castration

## Abstract

Few studies address behavioral changes after deslorelin treatment in cats. As increasingly more owners opt for pharmacological sterilization, understanding deslorelin effects on behavior is critical. This multicentric study compared behavioral changes in cats that were surgically sterilized with those that were treated with deslorelin using an anonymous survey of 19 behavioral changes. Owners of deslorelin-implanted cats completed two questionnaires related to the flare-up and downregulation periods, while owners of gonadectomized cats completed only one questionnaire. A total of 66 answers were collected (24 for surgical sterilization, 24 for flare-up, and 18 for downregulation). Decrease in urine marking, excessive vocalization, intact male cat urine odor, and disobedience were significantly higher in downregulated cats compared to gonadectomized ones. In the flare-up group, 68% of females exhibited behavioral manifestation of induced heat. Males displayed flare-up behaviors less frequently and intensively than females after implantation with deslorelin. Age at sterilization exerted a significant effect on five behaviors in deslorelin-implanted cats. Owners of deslorelin treated cats in the downregulation phase reported a more pronounced decrease in undesirable behaviors than the owners of surgically sterilized cats.

## Simple Summary

Sterilization, whether surgical or pharmacological, induces physiological and behavioral changes in cats. In this study, we compared behavioral changes in surgically sterilized and deslorelin-implanted cats through questionnaires sent to owners. We analyzed the frequency and intensity of nineteen behavioral and clinical changes after surgical sterilization and during two distinct phases of deslorelin treatment: flare-up and downregulation. We found that female cats display some behavioral changes during flare-up more noticeably than male cats. The expression of several behaviors was also influenced by age. Owners of deslorelin-implanted cats reported a significant decline in negatively perceived behaviors during downregulation, compared with owners of surgically sterilized cats.

## 1. Introduction

Reproduction control plays a crucial role in veterinary medicine as it contributes to animal welfare by preventing overpopulation and controlling roaming, thereby decreasing the risk of injuries due to accidents and fights as well as the spreading of diseases [1,2]. Surgical gonadectomy, the most common option for reproduction control of cats, has come under close scrutiny in recent years [1]. Pet owners increasingly question efficacy and safety of this procedure due to both unfounded concerns about surgery and anesthesia, as well as awareness regarding potential long-term effects [2,3,4], such as urinary incontinence [5], increased incidence of neoplastic diseases [6], obesity [7,8] and orthopedic diseases [9]. A recent preliminary study suggests that, similarly to dogs, surgical gonadectomy may increase the overall risk of tumor development in cats, and that age at the time of the procedure may play a crucial role [10].

Consequently, pharmacological sterilization is increasingly being considered as a temporary alternative solution, particularly by cat breeders, as it allows them to comply with current international regulations of feline breeding associations [11], which limit cats to producing no more than three litters per year. Pharmacological sterilization can currently be achieved using subcutaneous implants of deslorelin, a synthetic long-lasting gonadotropin releasing hormone (GnRH) agonist, which is 100 times more potent than natural GnRH [12,13]. Deslorelin is the only marketed long-acting GnRH agonist for inducing temporary infertility in male [14] and pre-pubertal female dogs [15], and since June 2022, also in male cats [16,17]. It is marketed in the form of a subcutaneous implant of 4.7 or 9.4 mg of the active ingredient deslorelin acetate, and its duration of action in cats is estimated to be at least twice as long as the corresponding duration in dogs, which is six and twelve months for the 4.7 and 9.4 mg implant, respectively [18].

Following deslorelin administration, the continuous release of the GnRH agonist will cause prolonged pituitary stimulation, leading to the release of luteinizing hormone (LH) and follicle-stimulating hormone (FSH) which may in some individuals cause clinical signs of excess libido and hyperactivity (in males) or estrus induction (in females treated in anestrus [19,20,21,22,23,24,25]). Incidence of these clinical signs (collectively indicated as “flare-up” reaction) in deslorelin-treated animals has not been defined.

After the initial stimulation of the reproductive axis, pituitary GnRH receptors are desensitized, resulting in suppressed secretion of FSH and LH and a blockade of gonadal hormones production [19,20,21,23,24,25,26]. The exact LH and FSH release patterns which occur during the process of downregulation have not been studied in cats [23]. Instead, the efficacy of deslorelin treatment has been reported in multiple studies through the use of indirect markers, such as testosterone and progesterone concentrations in peripheral blood and estradiol concentrations in the feces [17,19,20,27,28]. The complete downregulation process in tomcats takes a minimum of 10 weeks and can extend up to 16 weeks [16,27,29] although periods up to 28 weeks have been reported [17]. Regardless of treatment duration [19,27,30], reversibility of the effect of deslorelin has been demonstrated with successful restoration of fertility in both male and female cats [30,31].

Behavioral changes in deslorelin-treated cats have only been reported as a parallel observation in trials with different aims [17,19,20,25,28,32]. One study reported changes due to the flare-up phase in male cats as an increase in reproductive behavior (libido, mounting, mating, and urine marking) persisting until the 16th day after implantation. After the initial flare-up phase, these unwanted behavior patterns diminished [17]. Similar behavioral changes, indicative of an estrous cycle, were reported in female cats during the flare-up phase [25]. Another randomized controlled trial also reported a significant reduction in reproductive behaviors, such as vocalization, urine marking, aggression, and increase in male cat urine odor in deslorelin treated compared to intact tomcats, with no behavioral change observed in the latter [16]. However, no study has focused on the comparison with surgically sterilized cats. Therefore, the purpose of this study was to compare the behavioral response of cats following pharmacological (administration of deslorelin) or surgical (gonadectomy) sterilization. Preliminary results of this study on demographic data and significant behavioral changes were published in the form of an abstract in a conference proceeding [33].

## 2. Materials and Methods

This multicentric study was conducted at two academic and one private veterinary hospital, the Clinic for Reproduction of the Veterinary Faculty of the University of Ljubljana, Slovenia, the Veterinary Teaching Hospital of the University of Padova, Italy, and the San Marco Veterinary Clinic in Veggiano, Italy, respectively. Cat owners provided their informed consent to the collection and analysis of the data in agreement with the General Data Protection Regulation (GDPR). In accordance with Directive 2010/63/EU on the protection of animals used for scientific purposes, and the European Commission Decision of 29 October 2012, extended by Protocol No. 02-2016, this study did not require formal ethics committee approval. Instead, it relied on questionnaires distributed to cat owners visiting each veterinary institution for standard examinations or treatments.

### 2.1. Study Population

The study population was composed of healthy post-pubertal cats presented for surgical or pharmacological sterilization. The owners of pharmacologically sterilized cats were requested to complete a questionnaire at two times during the treatment: the first two weeks after implantation, i.e., a “flare-up” (FU) questionnaire, and three months after implantation, i.e., a “downregulation” (DR) questionnaire. Owners of surgically sterilized (SS) cats answered another questionnaire, which was sent to them at least three months after the surgical procedure. Only animals whose sterilization procedure (pharmacological or surgical) was performed less than two years prior to answering the questionnaire were included in the study.

### 2.2. Data Collection

A link to a Google Form questionnaire in Italian, Slovenian, or English was sent to the owners by e-mail. All owners answered anonymously. To ensure clarity and consistency in the interpretation of behavioral terms, a document defining each behavior (available in the three different languages) was included in the e-mail (see Supplementary Materials). The same set of questions was used for all groups, with the only variation being the specific time point after (pharmacological or surgical) sterilization, which owners were asked to consider when evaluating their cat behavioral changes. Each questionnaire was divided into three sections: animal data (basic details such as the cat’s sex, age, breed, and date of sterilization); living conditions (interactions of treated cats with other animals and people in the household, as well as their living environment, including whether they had access to outdoors); and behavioral and clinical changes (changes recorded during the current phase of treatment, whether flare-up, downregulation, or post-surgical sterilization). Nineteen behavioral and clinical changes potentially correlated with the presence or absence of reproductive hormones were considered: *reproductive behavior* (e.g., mounting attempts in males, lordotic position and acceptance of mounting in females, see Supplementary Materials for further details), *fearfulness*, *urine marking*, *inadequate urination*, *inadequate defecation*, *intact male cat urine odor*, *roaming*, *disobedience*, *destructive behavior towards objects*, *intra-species aggressiveness not associated with play*, *inter-species aggressiveness not associated with play*, *aggressiveness towards people not associated with play*, *affection towards the owner*, *attention seeking*, *excessive vocalization*, *excessive hair grooming*, *inadequate hair grooming*, *physical activity*, and *appetite*. These were rated on a Likert scale in relation to the cat behavior before and during treatment. A scale of 1 to 3 was used to measure intensity and frequency with “decrease”, “no alteration”, and “increase” being labelled as 1, 2, and 3, respectively. This scale also included a “not applicable” option. We considered a change in behavior as relevant when more than 30% of owners indicated an increase or decrease in this characteristic in any of the three groups (e.g., more than 30% (0.3) of owners in the FU group rated the change in *reproductive behavior* as an increase). The final section of the questionnaire assessed whether any alterations in the owner’s or animal’s routine (e.g., introduction of a new animal, relocation) coincided with the timing of implant administration and could have influenced the animal’s behavior. Respondents who reported such changes were asked to provide a detailed description.

Age at sterilization was calculated from the date of sterilization and the cat’s age at the time of answering the questionnaire. Cats were categorized into three age groups: group 1 (young cats) included cats up to one year, group 2 (young-adult cats) included cats between one and three years of age, and group 3 (adult cats) included cats older than three years.

Flare-up behavior was defined as either an increase in *reproductive behavior* of 50% or more, or an increase in *reproductive behavior* of less than 50% associated with an increase in two or more of the following behaviors: *urine marking*, *intact male cat urine odor*, *roaming*, and *excessive vocalization*. Only FU questionnaire responses related to behaviors within the first two weeks following deslorelin administration were considered for estimating the rate of expression of flare-up behavior.

### 2.3. Sterilization Procedures

All selected cats for this study underwent a thorough general clinical examination as well as a reproductive exam which, in queens, included palpation of the mammary glands, transabdominal palpation of the uterus, vaginal cytology (to rule out estrus), and uterine ultrasound, and in tomcats included palpation of the testis and unsheathing the prepuce for detection of penile spikes. All surgically sterilized cats underwent routine bilateral orchiectomy and ovariectomy, while pharmacologically sterilized cats received a 4.7 mg or 9.4 mg deslorelin implant (Suprelorin^TM^ Virbac, Carros, France). The implant was inserted subcutaneously in either the interscapular or umbilical region. After insertion of the implant, the cats were not examined again unless this was necessary or requested by the owner. After surgical sterilization, both female and male cats were routinely checked approximately one week to ten days later to assess suture integrity and overall recovery of the patient.

### 2.4. Statistical Analysis

All questionnaires were analyzed and related data entered into Microsoft Excel. Descriptive statistics were performed to provide a comprehensive overview of experimental groups. The proportions of purebred versus mixed breed cats, age at sterilization below or above one year-old and indoor or outdoor access status across groups (FU, DR, and SS) were analyzed with both a Pearson’s chi-square test and a chi-square test for likelihood ratio [34]. Both results are presented. The comparison between behavioral/clinical changes in the DR and SS groups was tested with a two-proportion z test (z-score). The age effect on the expression of the 19 behavioral/clinical changes in the three different groups was assessed with the Kruskal–Wallis statistical test. The correlation between changes in different behaviors was tested with a Spearman’s rank correlation test. The correlation values (r_s_) were interpreted as follows: 0.00 – |0.30|: negligible correlation; |0.30| – |0.50|: low correlation; |0.50| – |0.70|: moderate correlation; |0.70| – |0.90|: high correlation; |0.90| – |1.00|: very high [28]. Not applicable answers were removed.

## 3. Results

A total of 66 questionnaires were completed of which 24, 24, and 18 were obtained from SS, FU, and DR cats, respectively. The response rates were 41.4% for the SS group, 61.5% for the FU group, and 52.9% for the DR group, based on the 58, 39, and 34 questionnaires sent to each group, respectively. As questionnaires were anonymous, it was impossible to understand whether or not a DR owner had already filled out a FU questionnaire. Therefore, FU and DR questionnaires were treated as if they were from different groups of cats. No owner reported about any event taking place concurrently to either SS or DR procedures (i.e., change in environment, introduction of a new animal to the house, change of diet, prolonged absence of one family member etc.), that could be responsible for the subsequently reported behavioral changes.

A higher proportion of cats in the SS group (0.63) were sterilized before reaching one year of age, compared to only 0.44 and 0.29 in the FU and DR groups. However, the difference between these proportions was not statistically significant with the likelihood ratio chi-square (*p* = 0.11) and neither with the Pearson’s (*p* = 0.061). Indoor living environments were more common among deslorelin-implanted cats, with 0.79 of FU and 0.89 of DR cats living indoors, compared to just 0.38 of the SS group. Although a statistically significant difference was found regarding the living environment across groups using the Pearson’s chi-square test (*p* < 0.001), the likelihood ratio chi-square test, which is more appropriate for smaller sample sizes and skewed proportions, yielded a non-significant result (*p* = 0.22). Mixed breed cats were more prevalent in the SS group (0.83), while purebred cats predominated in the FU (0.75) and DR (0.83) groups. The proportion of mixed breed cats in the SS group was considered significantly different from the FU and DR groups’ in both the likelihood ratio (*p* ≈ 0.05—marginally significant) and the Pearson’s (*p* < 0.001) chi-square test. Additional data regarding age at sterilization, living environment, and breed distribution among the groups are presented in Table 1.

The most relevant behavioral/clinical changes in the SS group were a decrease in *reproductive behavior* (0.58) and an increase in *affection towards the owner* (0.50), *attention seeking* (0.42), *appetite* (0.42), and *physical activity* (0.38). The remaining values and proportions of the behavioral/clinical changes in the SS group are presented in Table 2.

The most relevant behavioral/clinical changes in the FU group were an increase in *excessive vocalization* (0.67), *affection towards the owner* (0.46), *attention seeking* (0.42), *disobedience* (0.38), *reproductive behavior* (0.33), *physical activity* (0.33), and *urine marking* (0.33). A relevant decrease in *appetite* (0.33) was also noticed. The remaining values and proportions of the behavioral/clinical changes in the FU group are represented in Table 3.

Eight female cats (33.3%) exhibited behavioral flare-up: four of them showed an increase > 50% in *reproductive behavior*, whereas the other four showed an increase in *reproductive behavior* ≤ 50% along with an increase in two or more of the following behavioral changes: *urine marking*, *roaming*, and *excessive vocalization.* None of the male cats in the FU group exhibited behavioral flare-up.

The most relevant behavioral/clinical changes in the DR group were a decrease in *reproductive behavior* (0.78), *urine marking* (0.50), *intact male cat urine odor* (0.39), *disobedience* (0.33), and *physical activity* (0.33), and an increase in *attention seeking* (0.67), *affection towards the owner* (0.61), *appetite* (0.44), and *fearfulness* (0.33). The remaining values and proportions of behavioral/clinical changes in the DR group are represented in Table 4.

### 3.1. Comparison Between the Downregulation (DR) and Surgical Sterilization (SS) Groups

In order to statistically compare the DR and SS groups, the proportions of the relevant behavioral changes (>0.3) were recalculated discarding the non-applicable answers (Table 5). The proportion of cats with decrease in *urine marking* in the DR group (0.64) was significantly higher (*p* = 0.026) compared to the SS group (0.29). The same trend was observed for *excessive vocalization* (*p* = 0.024), *intact male cat urine odor* (*p* = 0.019), and *disobedience* (*p* = 0.007). Furthermore, no cat in the DR group exhibited an increase in *intact male cat urine odor*, whereas two in the SS group did. Regarding *disobedience*, a higher proportion of cats in the SS group exhibited an increase (0.24) compared to those showing a decrease (0.10). The proportion of cats with decrease in *physical activity* was not significantly different in the DR and SS groups. However, an increase in *physical activity* was significantly higher in the SS group (*p* = 0.005). Also, the share of cats who manifested an increase in *attention seeking* was significantly higher in the DR group (*p* = 0.005).

Absence of statistically significant differences between proportions in the SS and DR groups were observed for decrease in *reproductive behavior* and increases in *fearfulness*, *affection towards owners,* and *appetite.*

### 3.2. Age Effect

A significant effect of age was observed in the FU group regarding the increase in *reproductive behavior*. Young-adult cats (one to three years old) were more likely to exhibit *reproductive behavior* within the first two weeks after deslorelin implantation, compared to cats older than three years (*p* = 0.012). Additionally, cats younger than one year were significantly less likely to display *intact male cat urine odor* (*p* = 0.0273) *and inadequate urination* (*p* = 0.047) during the FU phase compared to older cats. *Appetite* was significantly increased in adult cats older than three years compared with younger cats in the FU group (*p* = 0.027). In the DR group, a significant age effect was also observed for *fearfulness* (*p* = 0.016), with young-adult cats (one to three years-old) being more prone to express fearful behavior compared to older cats.

### 3.3. Behavioral Changes Correlation

A significant positive correlation was identified across all three groups (r_s_ and *p*-values, respectively, for SS, FU, and DR) between *intra-species aggressiveness* and *inter-species aggressiveness* (r_s_ = 0.602, *p* < 0.01; r_s_ = 0.758, *p* < 0.01; r_s_ = 0.8, *p* < 0.01), and *inter-species aggressiveness* and *aggressiveness towards people* (r_s_ = 0.681, *p* < 0.01; r_s_ = 1, *p* = 0—removing the non-applicable answers, all answers were equal; r_s_ = 1, *p* = 0—removing the non-applicable answers, all answers were equal). A positive correlation was observed between *intra-species aggressiveness* and *aggressiveness towards people* across all groups; however, it was only statistically significant in the FU and DR groups (r_s_ = 0.613, *p* < 0.01; r_s_ = 0.775, *p* = 0.024), while in the SS group the correlation was positive but not significant (r_s_ = 0.352, *p* = 0.181). *Roaming* and *reproductive behavior* showed a significant positive correlation only in the FU group (r_s_ = 0.728, *p* < 0.01). For *inadequate urination* and *inadequate defecation*, significant positive correlation was observed in all groups (r_s_ = 0.858, *p* < 0.01; r_s_ = 0.558, *p* < 0.01; r_s_ = 0.748, *p* < 0.01). *Appetite* and *physical activity* were negatively correlated, although not significantly, in the DR and FU groups, while, surprisingly, in the SS group, a significant low positive correlation was observed (r_s_ = 0.499, *p* = 0.018).

## 4. Discussion

The purpose of this study was to evaluate and compare owner-reported behavioral changes in cats treated with deslorelin implant and in cats undergoing surgical sterilization.

Our study revealed distinct differences in the timing and methods of sterilization between the SS group and the deslorelin-implanted groups, likely influenced by both owner preferences and veterinary recommendations. More than 50% of the cats in the SS group were surgically sterilized before one year of age, while the majority of deslorelin-implanted cats underwent sterilization after their first year. This finding suggests that the age of the cat plays a role in the owner’s decision-making process regarding sterilization methods. These results indicate that owners of cats under one year of age predominantly opted for surgical sterilization, likely driven by the intention to prevent reproduction and mitigate behaviors associated with puberty. In contrast, for cats older than one year, deslorelin implants were more frequently chosen as they are non-permanent solution to suppress undesirable reproductive behaviors during the breeding season without compromising future reproductive potential. This trend may partially reflect veterinary guidance, particularly for non-breeding cats, where surgical sterilization is often recommended as a definitive solution. Conversely, for purebred cats intended for breeding, owners may be advised to maintain reproductive integrity, leading to a preference for the reversible effects of deslorelin implants. The reversibility of the deslorelin implants offers advantages for breeders allowing for a temporary suppression of reproductive activity in cats that are not intended for breeding during a particular time period. Most importantly, deslorelin implants enable breeders to manage the production of litters by breeding queens, while adhering to current international requirements [11]. Once a cat reaches the end of its breeding life, surgical sterilization is generally the preferred option.

In our study, 61.5% of the queens in the FU group showed behaviors consistent with induced heat, resulting from an increase in reproductive hormones during the flare-up phase. While this percentage is notable, the fact that our results were based only on behavioral assessments provided by the owners is certainly a limitation. As silent heats may occur in queens, performing vaginal cytology would have produced more reliable results. Additionally, the occurrence of a flare-up phase in queens is closely linked to the reproductive stage at which they receive the implant. Queens implanted during anestrus or interestrus are more likely to exhibit flare-up behavior following treatment, opposed to queens implanted during diestrus [19,25]. Conversely, none of the 11 males in the FU group met the criteria for flare-up behavior. Again, this does not mean that an increase in serum testosterone did not occur, only that it did not result in obvious behavioral changes. Based on our observations, it appears that feline behaviors typical of the flare-up phase may be more easily noticed in females than in males.

When comparing the behaviors between SS and DR cats, it is important to highlight that cats in the DR group exhibit a significantly more pronounced decline in negatively perceived behaviors (*urine marking*, *intact male cat urine odor*, *excessive vocalization*, and *disobedience*) compared to surgically sterilized ones. Even though the sample sizes between the two groups are different, it is evident that owners in the DR group reported a greater decrease in the above-mentioned behaviors, compared to surgical sterilization. This result may be biased by the fact that owners of cats experiencing a flare-up noticed a more evident reduction of the negatively perceived behaviors. In spite of the fact that owners were instructed to assess their cats’ behavioral changes during the flare-up and downregulation phases independently, there may still have been a tendency to compare the two phases directly. Furthermore, cats with outdoor access (predominantly belonging to the SS group) may have been observed for a shorter period of time by their owners, and therefore some of their behavioral changes, mainly the ones related to elimination (*inadequate defecation* and *inadequate urination*) may have gone unnoticed. Although all cats were post-pubertal at the time of sterilization, more than half of the SS cats underwent surgery prior to one year of age; this may have led owners to overlook behavioral changes because such behaviors were not perceived as remarkably intense or frequent before the surgery.

All of the pharmacologically sterilized cats in this study were reproductively active prior to implantation. Some of them had previously been implanted with deslorelin, yet the exact number of re-implanted cats remains unknown due to the anonymity of the questionnaire and its lack of this specific inquiry. Owners who chose to re-implant their cats are likely satisfied with the effects of deslorelin. Considering the variability in individual responses to deslorelin, it is plausible that the DR group may overrepresent cats that responded particularly well to the treatment. This could have contributed to the encouraging results observed in the DR group. While these results cannot be generalized to the wider population, it is possible that owners might consider deslorelin more favorably than surgical sterilization when it comes to behavioral improvement.

When comparing our findings with the current literature [30], it is crucial to recognize the differences in study methodologies, including variations in scoring, behavioral definitions and observation periods, which limit comparability. In one study [16], although a flare-up phase was not clearly defined, the increase in reproductive behaviors until day seven suggests that a flare-up may have occurred. A continuous decrease in reproductive behavior score (including a decrease in vocalization, urine marking, urine odor, and aggression) was reported from day seven onwards [16], being consistent with the decrease in *reproductive behavior* (defined as an attempt of mounting in males and lordotic position in females) in the downregulation period found in our study.

The finding of five age effects suggests that behavioral responses to sterilization may vary depending on the age at which the procedure is performed. Specifically, young-adult cats implanted with deslorelin were more likely to exhibit *reproductive behavior* during the FU phase compared to older cats, suggesting increased behavioral sensitivity to high levels of GnRH at this age. Similarly, young deslorelin-treated cats less than one year of age were less likely to exhibit increased *intact male cat urine odor*, or *inadequate urination* during the first two weeks after implantation. Also, increases in *appetite* were more pronounced in adult cats in the FU group. In the DR group, *fearfulness* was expressed differently depending on the age at implantation. Our sample size is quite small, which may question the test power and validation for extrapolation of the results into the general population. However, unveiling an effect of age on behavioral changes after (pharmacological) sterilization supports the notion that age plays a critical role in how cats respond to sexual hormone increment (FU) and withdrawal (DR). Similar patterns have been observed in a study) in which dogs less than 3 years-old showed hypersexuality more frequently than older dogs [35]. This study also showed that improvement in aggressive behavior following treatment with Gonazon was more pronounced in younger dogs compared to older ones [35]. This may be due to a “learning effect” as it happens with surgical sterilization: behavioral response decreases with time elapsed between acquiring the behavior and sterilization [36]. Similarly, young cats may manifest less evident behaviors during the FU phase, e.g., inadequate urination, compared with older cats, as these animals had not yet developed these behaviors prior to implantation. Overall, these age-effect findings suggest that the timing of sterilization could influence the degree and nature of behavioral changes in cats, making it an important consideration for both veterinarians and cat owners when deciding on the method and timing of sterilization. Further research with larger sample sizes is needed to confirm these age-related patterns and to better understand how sterilization affects feline behavior at different ages.

In contrast to our expectations, a significant positive correlation between *appetite* and *physical activity* was found in SS (but not FU and DR) cats. Also, the proportion of cats who exhibited increase in *physical activity* was significantly higher in the SS group (0.41) than in the DR group (0.06). This raises questions regarding the timing of onset of alteration in metabolism, physical activity and appetite following sterilization in cats. Prior evidence has shown that in surgically sterilized cats, increased food intake and decreased physical activity occur two days and 12 weeks following surgery, respectively [37,38]. Our results suggest that a reduction in physical activity may require more time in surgically sterilized cats than in cats implanted with deslorelin (once the FU is over). During the time the SS questionnaire was completed, most cats showed increases in both *appetite* and *physical activity* which is not what would be expected of (medically and surgically) sterilized cats (*appetite* increasing and *physical activity* decreasing). However, we believe that this unexpected result may be more due to a mismatching on the time of answering the questionnaire (owners answered the questionnaire at least three months after sterilization, but no limit date was imposed), than to a real different metabolic effect arising from pharmacological or surgical sterilization. Although both the SS and DR groups gathered responses at least three months post-sterilization, the exact time between sterilization and answering the questionnaire varied widely among individuals, ranging from 90 to 710 days, which poses a risk of memory recall bias typical of questionnaire-based retrospective studies [39,40].

Another limitation of our study is the impossibility to know whether the owners who answered the FU questionnaire had already answered the DR questionnaire. As a consequence, the extent to which cats in the DR and FU overlap is unknown. The lack of a homogenous group of owners with deslorelin-implanted cats who completed both the FU and DR questionnaires makes it impossible to assess incidence of the FU reaction in implanted animals. Also, a question about the implant’s dosage (4.7 or 9.4 mg) was not included in the questionnaire, which constitutes a shortcoming, as it was not possible to assess whether dosage may exert some effect on behavioral response. Notwithstanding, there is no evidence in the literature that indicates that it may be the case. To date, the duration of effect of the implant is the only dependent outcome of deslorelin dosage [19,24,30]. It is also worth noting that, as is the case of all questionnaire-based surveys, behavior was not assessed directly, but by proxy. As our results are based on owners’/caretakers’ perception of their cat’s behavior, it is possible that differences in knowledge, attitudes, and sensibility may influence the assessment of treatment effect. The intrinsic limitations of surveys are also likely to apply at least to some extent [41].

## 5. Conclusions

Female cats tend to display more noticeable flare-up behaviors compared to males after implantation with deslorelin. Male cats showed minimal behavioral changes during the first two weeks after implantation, despite likely increases in testosterone, suggesting that behavioral flare-up is milder in this sex. Owners of deslorelin-implanted cats reported a more pronounced decline in undesirable behaviors during the downregulation phase compared to owners of surgically sterilized cats, which may be due to a higher degree of satisfaction with pharmacological sterilization when it comes to behavioral improvement. Additionally, deslorelin implants were more commonly used by owners of purebred and indoor cats, likely due to their breeding potential and reversibility of the implant. Finally, age-related effects on behavior, particularly in younger cats, suggest that sterilization timing could influence behavioral outcomes. Further research with larger sample sizes is needed to explore this correlation and better understand the behavioral impacts of sterilization across different age groups and methods.

## Figures and Tables

**Table 1:** Distribution of number (n) and proportion (∝) of cats by sex, age, breed, and living environment across the three groups (surgical sterilization, flare-up, and downregulation).

	Sex n (∝)		Age at the Time of Sterilization n (∝) ^a^			Breed n (∝)		Living Environment n (∝)	
	Female	Male	<1 Year	1–3 Years	>3 Years	Mixed Breeds ^b^	Pure Breeds	Indoor	With Outdoor Access
Surgical sterilization group (N = 24)	16 (0.67)	8 (0.33)	15 (0.63)	8 (0.33)	1 (0.04)	20 (0.83) *	4 (0.17)	9 (0.375)	15 (0.625)
Flare–up group (N = 24)	13 (0.54)	11 (0.46)	10 (0.44)	7 (0.30)	6 (0.26)	6 (0.25) °	18 (0.75)	19 (0.79)	5 (0.21)
Downregulation group (N = 18)	9 (0.50)	9 (0.50)	5 (0.29)	9 (0.53)	3 (0.18)	3 (0.17) °	15 (0.83)	16 (0.89)	2 (0.11)

^a^ One subject in FU and DR groups was excluded from age at sterilization analysis due to incorrect data provided by the responder. ^b^ Includes European shorthair. * and ° indicate statistical significance between groups with likelihood ratio chi-square test.

**Table 2:** Number (n) and proportion (∝) of cats whose 19 reproductive hormone related behaviors decreased (1), did not change (2), or increased (3) 3 months after surgical sterilization (surgical sterilization group).

Behavioral/Clinical Change	(1) Decrease		(2) No Changes		(3) Increase		Not Applicable	
	n	∝	N	∝	n	∝	n	∝
Reproductive behavior	**14**	**0.58**	4	0.17	1	0.04	5	0.21
Fearfulness	3	0.13	15	0.63	6	0.25	0	0
Urine marking	5	0.21	11	0.46	1	0.04	7	0.29
Inadequate urination	4	0.17	10	0.42	1	0.04	9	0.38
Inadequate defecation	1	0.04	11	0.46	1	0.04	11	0.46
Intact male cat urine odor	3	0.13	5	0.21	2	0.08	14	0.58
Roaming	5	0.21	8	0.33	4	0.17	7	0.29
Disobedience	2	0.08	14	0.58	5	0.21	3	0.13
Destructive behavior towards objects	2	0.08	17	0.71	2	0.08	3	0.13
Intra-species aggressiveness not associated with play	2	0.08	13	0.54	4	0.17	5	0.21
Inter-species aggressiveness not associated with play	2	0.08	12	0.50	5	0.21	5	0.21
Aggressiveness towards people not associated with play	3	0.13	13	0.54	3	0.13	5	0.21
Affection towards the owner	4	0.17	8	0.33	**12**	**0.50**	0	0
Attention seeking	4	0.17	10	0.42	**10**	**0.42**	0	0
Excessive vocalization	7	0.29	13	0.54	1	0.04	3	0.13
Excessive hair grooming	2	0.08	16	0.67	4	0.17	2	0.08
Inadequate hair grooming	2	0.08	16	0.67	0	0	6	0.25
Physical activity	4	0.17	9	0.38	**9**	**0.38**	2	0.08
Appetite	2	0.08	12	0.50	**10**	**0.42**	0	0

Values in **bold** represent relevant behavioral/clinical changes (>0.3 increase or decrease).

**Table 3:** Number (n) and proportion (∝) of cats whose 19 reproductive hormone related behaviors decreased (1), did not change (2), or increased (3) during first 2 weeks after pharmacological sterilization (flare-up group).

Behavioral/Clinical Change	(1) Decrease		(2) No Changes		(3) Increase		Not Applicable	
	N	∝	n	∝	n	∝	n	∝
Reproductive behavior	4	0.17	10	0.42	**8**	**0.33**	2	0.08
Fearfulness	2	0.08	19	0.79	3	0.13	0	0
Urine marking	2	0.08	11	0.46	**8**	**0.33**	3	0.13
Inadequate urination	3	0.13	13	0.54	**7**	**0.29**	1	0.04
Inadequate defecation	1	0.04	19	0.79	2	0.08	2	0.08
Intact male cat urine odor	4	0.17	5	0.21	5	0.21	10	0.42
Roaming	1	0.04	10	0.42	6	0.25	7	0.29
Disobedience	5	0.21	9	0.38	**9**	**0.38**	1	0.04
Destructive behavior towards objects	5	0.21	13	0.54	4	0.17	2	0.08
Intra-species aggressiveness not associated with play	1	0.04	14	0.58	3	0.13	6	0.25
Inter-species aggressiveness not associated with play	1	0.04	13	0.54	4	0.17	6	0.25
Aggressiveness towards people not associated with play	2	0.08	14	0.58	4	0.17	4	0.17
Affection towards the owner	5	0.21	8	0.33	**11**	**0.46**	0	0
Attention seeking	4	0.17	9	0.38	**10**	**0.42**	1	0.04
Excessive vocalization	1	0.04	4	0.17	**16**	**0.67**	3	0.13
Excessive hair grooming	0	0	13	0.54	7	0.29	4	0.17
Inadequate hair grooming	1	0.04	18	0.75	1	0.04	4	0.17
Physical activity	1	0.04	13	0.54	**8**	**0.33**	2	0.08
Appetite	**8**	**0.33**	10	0.42	5	0.21	1	0.04

Values in **bold** represent relevant behavioral/clinical changes (>0.3 increase or decrease).

**Table 4:** Number (n) and proportion (∝) of cats whose 19 reproductive hormone related behaviors decreased (1), did not change (2), or increased (3) three months after pharmacological sterilization (downregulation (DR) group).

Behavioral/Clinical Change	(1) Decrease		(2) No changes		(3) Increase		Not Applicable	
	n	∝	n	∝	n	∝	n	∝
Reproductive behavior	**14**	**0.78**	1	0.06	2	0.11	1	0.06
Fearfulness	3	0.17	6	0.33	**6**	**0.33**	3	0.17
Urine marking	**9**	**0.50**	4	0.22	1	0.06	4	0.22
Inadequate urination	5	0.28	6	0.33	4	0.22	3	0.17
Incomplete defecation	1	0.06	8	0.44	2	0.11	7	0.39
Intact male cat urine odor	**7**	**0.39**	2	0.11	0	0	9	0.50
Roaming	4	0.22	3	0.17	0	0	11	0.61
Disobedience	**6**	**0.33**	6	0.33	1	0.06	5	0.28
Destructive behavior towards objects	4	0.22	7	0.39	1	0.06	6	0.33
Intra-species aggressiveness not associated with play	5	0.28	4	0.22	1	0.06	8	0.44
Inter-species aggressiveness not associated with play	4	0.22	5	0.28	0	0	9	0.50
Aggressiveness towards people not associated with play	4	0.22	7	0.39	0	0	7	0.39
Affection towards the owner	2	0.11	4	0.22	**11**	**0.61**	1	0.06
Attention seeking behavior	1	0.06	4	0.22	**12**	**0.67**	1	0.06
Excessive vocalization	10	0.56	2	0.11	3	0.17	3	0.17
Excessive hair grooming	2	0.11	9	0.50	1	0.06	6	0.33
Inadequate hair grooming	3	0.17	10	0.56	0	0	5	0.28
Physical activity	**6**	**0.33**	11	0.61	1	0.06	0	0
Appetite	3	0.17	6	0.33	**8**	**0.44**	1	0.06

Values in **bold** represent relevant behavioral/clinical changes (>0.3 increase or decrease).

**Table 5:** Number (n) and proportion (∝) of cats with relevant changes in behaviors (>0.3) in surgical sterilization (SS) and downregulation (DR) groups. Denominator used for calculation of proportions (∝) corresponds to the difference between total number of cats in each group (SS and DR) and number of non-applicable (NA) answers, represented in the table as the Total columns.

	SS Group					DR Group				
Behavioral/Clinical Change	(1) Decrease		(3) Increase		Total ^a^	(1) Decrease		(3) Increase		Total ^a^
	N	∝	N	∝	n	n	∝	n	∝	n
Reproductive behavior	14	0.74	1	0.05	19	14	0.82	2	0.12	17
Fearfulness	3	0.13	6	0.25	24	3	0.20	6	0.40	15
Urine marking	5	0.29 *	1	0.06	17	9	0.64 °	1	0.07	14
Intact male cat urine odor	3	0.30 *	2	0.20	10	7	0.78 °	0	0	9
Disobedience	2	0.10 *	5	0.24	21	6	0.46 °	1	0.08	13
Affection towards the owner	4	0.17	12	0.50	24	2	0.12	11	0.65	17
Attention seeking	4	0.17	10	0.42 *	24	1	0.06	12	0.71 °	17
Excessive vocalization	7	0.33 *	1	0.05	21	10	0.67 °	3	0.20	15
Physical activity	4	0.18	9	0.41 *	22	6	0.33	1	0.06 °	18
Appetite	2	0.08	10	0.42	24	3	0.18	8	0.47	17

^a^ Number of responses excluding “Not applicable” answers. * and ° indicate statistical significance between the groups.

## Data Availability

Data are available from the authors upon request.

## Supplementary Materials

The following supporting information (definitions of behaviors provided to owners in English) can be downloaded at: https://www.mdpi.com/article/10.3390/vetsci12050430/s1, Document S1: Behavioural changes after sterilization–Backtranslation. The google forms questionnaires can be accessed at: https://forms.gle/ZfDEJz7ZkRD3u7Zx9 (SS-URL accessed on 27 April 2025), https://forms.gle/xw6DMTCUT4diSmvH6 (FU-URL accessed on 27 April 2025) and https://forms.gle/z4MSALtA3uZiF3v6A (DR-URL accessed on 27 April 2025).

## Abbreviations

The following abbreviations are used in this manuscript:

SS	Surgical sterilization
FU	Flare-up
DR	Downregulation
