# Supplementary material for: Behavioral Changes in Cats Following Deslorelin Implantation or Surgical Gonadectomy
Source: Vet Sci. 2025 Apr 30;12(5):430. doi: 10.3390/vetsci12050430 (PMC12115999; doi:10.3390/vetsci12050430)
Supplement: Supplementary file 1 [file vetsci-12-00430-s001.zip › vetsci-3536427-supplementary.pdf]

Behavioural changes after sterilization

Backtranslation

Sexual Behaviour

Female: In the presence of males or after petting of the lower back and neck, the female exhibits a lordotic position (lying the stomach on the ground maintaining the behind and hind limbs slightly elevated) and lateral movement of the tail. Acceptance of mounting from the male.

Male: Mounting attempts on females or objects.

Fearfulness

Escape or hide seeking in the face of something different or unknown to their environment (people, animals or objects).

Inadequate elimination

Urine marking: deposition of small quantities of urine in jet (spraying), specially on vertical surfaces with the animal standing, without lowering the hind limbs.

Inadequate urination: deposition of urine in horizontal surfaces, apart from their litter box, with the animal in a crouching position lowering the hind limbs.

Inadequate defecation: deposition of faeces in horizontal surfaces, apart from their litter box, with the animal in a crouching position lowering the hind limbs.

Intact male cat urine odour

Strong and pungent odour of male cat and/or urine.

Roaming

Tendency to escape home in order to find the opposite sex.

Disobedience

Cat behaviour contrary to the owner’s instructions, lack of discipline.

Destructive behaviour towards objects

Chewing or scratching objects.

Intra-species aggressiveness not associated with play

Aggressiveness towards other cats not in association with playing. Fighting, scratching and biting.

Inter-species aggressiveness not associated with play

Aggressiveness towards other animals that are not cats not in association with playing. Fighting, scratching and biting.

Aggressiveness towards people not associated with play

Aggressiveness towards people not in association with playing. Fighting, scratching and biting.

Affection towards the owner

Rubbing of the head, neck against the owner's legs, maintaining the tail straight, rubbing the muzzle against the owner's face, squinting, and purring while being cuddled.

Attention seeking

Miawning, walk between the owner's legs, trying to be near the owner.

Excessive vocalization

More frequent vocalizations and miawnings than the usual ones.

Excessive hair grooming

Excessive hair licking.

Inadequate hair grooming

Inconsistent and insufficient hair licking.

Physical activity

Jumping, climbing, running, and chasing objects or other animals.

Appetite

Eating with avidity.
